# A Diagnostic Dilemma: Abdominal Aortic Dissection Masquerading as Pyelonephritis

**DOI:** 10.1155/carm/6694346

**Published:** 2025-10-13

**Authors:** Elahe Nasri Nasrabadi, Keihan Golshani, Mehdi Nasr Isfahani

**Affiliations:** ^1^Department of Emergency Medicine, School of Medicine, Isfahan University of Medical Sciences, Isfahan, Iran; ^2^Department of Health in Disasters and Emergencies, School of Management and Medical Informatics, Isfahan University of Medical Sciences, Isfahan, Iran

**Keywords:** abdominal aortic dissection, CT angiography, pyelonephritis

## Abstract

This report underscores the urgent necessity of early and accurate diagnosis in cases of aortic dissection (AD) with atypical presentations. A 76-year-old male presented to Al-Zahra Hospital in Isfahan, Iran, with a 7-day history of left flank pain radiating to the left testicle and groin, accompanied by fever, hematuria, and dysuria. Initial assessments, including a kidney and urinary tract ultrasound, revealed severe left kidney hydronephrosis and free fluid, leading to a preliminary diagnosis of pyelonephritis with a possible abscess. Broad-spectrum antibiotics were promptly administered. However, a subsequent CT scan revealed a retroperitoneal hematoma, prompting a CT angiography (CTA), which confirmed an abdominal AD. Despite rapid surgical intervention, the patient succumbed to cardiac arrest. This case highlights the critical importance of vigilance and thorough investigation in patients with atypical symptoms to prevent potentially fatal misdiagnoses.

## 1. Introduction

Aortic dissection (AD) is a medical emergency that occurs when a tear develops in the inner lining of the aorta. This tear allows blood to enter and separate the layers between the aortic intima and media, creating a false lumen and increasing intraluminal pressure [1].

This dangerous event typically involves the thoracic aorta and is rarely limited to the abdominal aorta. Abdominal AD occurs in less than 2% of cases, whereas dissections of the ascending aorta, descending aorta, and aortic arch account for approximately 70%, 20%, and 7% of cases, respectively [2].

AD most commonly affects older men with a long history of hypertension. In addition, factors such as smoking, high cholesterol, a personal or family history of aortic disease, valvular heart disease, previous heart surgeries, sudden direct chest trauma, and the use of certain injectable drugs are associated with its onset [1, 3].

Differential diagnoses for AD include myocardial infarction (MI), pericarditis, myofascial pain syndrome, pulmonary embolism, chest abscess or severe lung inflammation, coronary artery spasm, aortic aneurysm, and genetic conditions such as Marfan syndrome or Ehlers–Danlos syndrome [2, 4].

Conversely, although pyelonephritis and AD are distinct diseases, they can sometimes present with similar clinical symptoms, complicating diagnosis. For example, due to its anatomical position, AD can affect the renal arteries, causing lower back and flank pain that mimics the symptoms of pyelonephritis [5].

Severe abdominal or lower back pain, changes in the patient's general condition (such as fever, chills, nausea, and vomiting), and alterations in blood pressure can manifest similarly in both conditions. However, specific characteristics of the pain, the presence of urinary symptoms, fever resulting from a urinary tract infection, internal bleeding, or secondary inflammation can help differentiate these diseases [6, 7].

Therefore, to accurately diagnose AD, it is essential to promptly assess clinical symptoms, distinguish similarities with other conditions, and perform imaging tests such as CT scans or echocardiography. This study presents a case of AD with unusual symptoms mimicking pyelonephritis.

## 2. Case Report

A 76-year-old male presented to Al-Zahra Hospital with a seven-day history of persistent left flank pain radiating to the left testicle and groin. His symptoms were accompanied by fever, dysuria, and a single episode of hematuria. He had previously sought care at a local medical center, where he was treated empirically for a presumed urinary tract infection over 3 days. However, due to a lack of clinical improvement, he self-referred to our facility for further evaluation.

The patient's medical history was notable for a recent low anterior resection (LAR) performed 1 month prior, a left lower limb amputation 30 years earlier, and longstanding hypertension. His current medications included allopurinol, diltiazem, levofloxacin, and rosuvastatin.

On physical examination, he was febrile and exhibited marked tenderness over the left costovertebral angle. The abdomen was soft and nondistended, and peripheral pulses in the upper extremities were full and symmetric. Given the clinical presentation and recent surgical history, renal and urinary tract ultrasonography was performed.

Sonographic evaluation revealed severe left-sided hydronephrosis and approximately 220 cc of perinephric free fluid. Internal echoes demonstrated septation, and the subcutaneous soft tissue of the medial left thigh exhibited a cobblestone appearance, suggestive of edema and inflammation. These findings, combined with laboratory results showing leukocytosis, elevated blood urea nitrogen (BUN) (36 mg/dL), and a serum creatinine level of 1.1 mg/dL, supported a preliminary diagnosis of pyelonephritis with possible abscess formation. Broad-spectrum antibiotic therapy was initiated, and a noncontrast CT scan of the abdomen and pelvis was ordered to further investigate the suspected infection.

**Figure 1:**
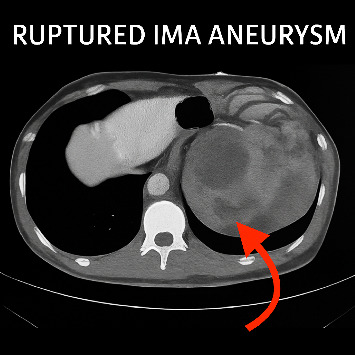
CT scan revealing ruptured IMA aneurysm with retroperitoneal hematoma (red arrow).

**Figure 2:**
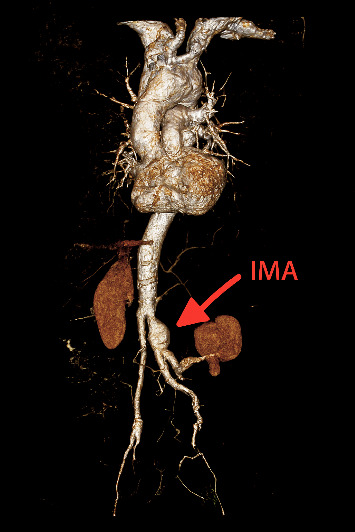
CTA 3D reconstruction showing ruptured IMA aneurysm and associated abdominal aortic dissection (red arrow).

Unexpectedly, the CT scan revealed a large retroperitoneal hematoma (Figure 1), prompting urgent CT angiography (CTA). The CTA confirmed the presence of a ruptured inferior mesenteric artery (IMA) aneurysm and an associated abdominal AD (Figure 2), establishing a vascular etiology for the patient's symptoms. An emergency surgical intervention had been planned; however, the patient experienced a sudden cardiac arrest prior to entering the operating theater, and resuscitation efforts were unsuccessful.

### 2.1. Chronology of Investigations

**Figure 3:**
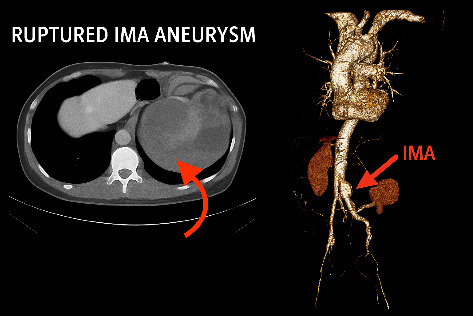
Axial CT and 3D CTA images demonstrating ruptured IMA aneurysm and associated aortic dissection (red arrows).

The clinical course unfolded over five critical days. During Days 1–3, the patient experienced persistent flank pain, fever, and urinary symptoms and was treated empirically at a local center. On Day 4, due to a lack of improvement, he presented to our hospital, where initial imaging and laboratory findings suggested pyelonephritis. Later that day, noncontrast CT imaging revealed a retroperitoneal hematoma, prompting reconsideration of the initial diagnosis. On the morning of Day 5, CTA confirmed a ruptured IMA aneurysm and AD (Figure 3). Tragically, the patient went into cardiac arrest later that afternoon, before surgical management could be initiated.

Although the initial ultrasound report suggested pyelonephritis or pyonephrosis, based on hydronephrosis and perinephric soft tissue changes, subsequent CT imaging did not reveal any renal abscess or parenchymal infection. Instead, the retroperitoneal hematoma was found to be compressing the left kidney, likely resulting in secondary hydronephrosis and mimicking an infectious process. Due to institutional protocols at the referring facility, the original ultrasound images were not archived or made available for retrospective review. Therefore, only the sonographic report findings are presented, limiting direct modality comparison.

## 3. Discussion

The aorta supplies blood to multiple organ systems along its course, and any disruption, whether due to thromboembolism or rupture, can result in organ damage that mimics other pathologies. This overlap in clinical presentation often leads to diagnostic confusion. In the present case, the patient's symptoms initially resembled pyelonephritis: left flank pain radiating to the testicle and groin, fever, dysuria, and hematuria. These findings, combined with ultrasound evidence of severe left-sided hydronephrosis, led to a preliminary diagnosis of a urinary tract infection. However, further imaging revealed a retroperitoneal hematoma, and CTA ultimately confirmed an abdominal AD, shifting the diagnostic paradigm.

Misdiagnosis in the emergency department is a well-documented challenge, particularly in cases of AD. Studies have reported initial misdiagnosis rates as high as 78% among patients later confirmed to have AD [6, 7]. In this case, the misleading constellation of urological symptoms, combined with supportive sonographic findings, delayed recognition of the underlying vascular pathology. The abdominal and pelvic CT scan played a pivotal role in identifying the retroperitoneal hematoma, which prompted further investigation via CTA and ultimately led to the correct diagnosis.

Fever, typically regarded as a hallmark of infection, can also be a misleading symptom in AD. In some instances, it reflects an inflammatory response to aortic wall injury rather than an infectious process [8]. Organ damage resulting from AD may manifest as abdominal pain, altered bowel habits, limb ischemia, stroke, acute kidney injury, congestive heart failure, or pleural effusion. Among these, pain remains one of the most reliable indicators of dissection [9].

The nature and location of pain in AD are closely tied to the anatomical site of the dissection. Ascending dissections often present with anterior chest, neck, or jaw pain, whereas descending dissections typically cause back or abdominal pain. Unlike MI, where pain builds gradually, AD pain is abrupt and reaches peak intensity immediately. It may also migrate, beginning in the chest and moving caudally. Although traditionally described as “tearing” or “ripping,” patients more frequently characterize the pain as sharp [4, 10].

Several case reports have highlighted the diagnostic pitfalls associated with atypical AD presentations. Ibraheem et al. described a case of Type B AD initially misdiagnosed as pyelonephritis, underscoring the importance of thorough history-taking, comprehensive physical examination, and early imaging. They advocated for the use of the AD Detection Risk Score to enhance diagnostic accuracy, particularly in emergency settings [6]. Similarly, Rane et al. reported a case of abdominal AD in a 28-year-old male who presented with fever and right flank pain. Despite antibiotic therapy, persistent symptoms prompted imaging that revealed a hematoma and abdominal AD with two ruptures. The patient ultimately died following surgical intervention due to delayed diagnosis [5].

Despite advances in diagnostic imaging and increased clinical awareness, the mortality rate associated with AD remains high, especially in cases of delayed recognition. Mortality increases by approximately 1% per hour during the first 24 h and can reach up to 50% by the third day [10]. This underscores the critical importance of early diagnosis and timely intervention.

The present case illustrates an isolated abdominal AD, an uncommon entity not fully encompassed by the Stanford or DeBakey classification systems, which primarily address thoracic involvement. While DeBakey Type IIIb dissections may extend into the abdominal aorta, they originate in the descending thoracic segment [11]. In contrast, this case involved a dissection confined entirely to the abdominal aorta. Given its atypical anatomical location and clinical presentation, such cases warrant distinct diagnostic consideration beyond traditional classification frameworks.

**Figure 4:**
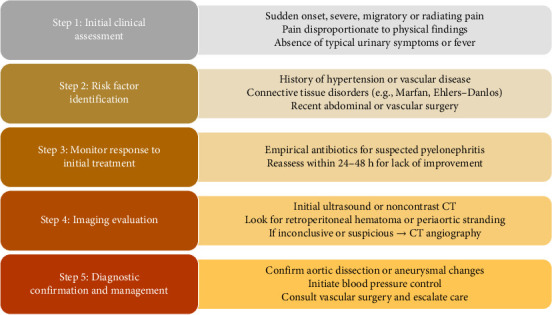
Hierarchical diagnostic algorithm for evaluating suspected vascular etiology in patients presenting with flank pain.

To aid clinicians in recognizing atypical presentations of abdominal AD, particularly when symptoms overlap with common urological conditions, we propose a hierarchical diagnostic algorithm (Figure 4).

### 3.1. Clinical Note

Early consideration of vascular etiologies in patients presenting with flank pain, particularly when accompanied by hemodynamic instability, atypical imaging findings, or poor response to antibiotics, can significantly enhance diagnostic accuracy and improve outcomes. CTA remains the gold standard for evaluating suspected AD and should be promptly employed when clinical suspicion persists.

## 4. Conclusion

This case highlights the critical importance of including AD in the differential diagnosis, even when patients present with symptoms that closely resemble other conditions, such as pyelonephritis. The atypical and deceptive nature of AD presentations can easily mislead clinicians, resulting in potentially fatal delays in diagnosis and treatment. Rapid and comprehensive initial imaging is essential to avoid these diagnostic pitfalls. By prioritizing timely diagnostic evaluation, healthcare providers can significantly improve diagnostic accuracy, expedite treatment, and enhance survival outcomes for patients with this life-threatening condition.

## Data Availability

The datasets used and/or analyzed during the present study are available from the corresponding author on reasonable request.

## Nomenclature

AD: Aortic dissection
CTA: CT angiography
CVA: Costovertebral angle
